# Wet etching of (−102) β-Ga_2_O_3_ with tetramethylammonium hydroxide (TMAH)

**DOI:** 10.1080/14686996.2026.2666988

**Published:** 2026-05-08

**Authors:** Takayoshi Oshima

**Affiliations:** Research Center for Electronic and Optical Materials, National Institute for Materials Science, Tsukuba, Japan

**Keywords:** β-Ga_2_O_3_, wet etching, TMAH

## Abstract

We investigated wet etching of (−102) β-Ga_2_O_3_ substrates using heated 25 wt% tetramethylammonium hydroxide (TMAH). The (−102) plane exhibited an etching rate that was one order of magnitude higher than those of the widely used (100), (010), (001), and (−201) orientations. In addition, the temperature dependence of the etching rate over the range 50°C–90°C was well described by the Arrhenius equation, with an activation energy that was nearly independent of carrier concentration. These two features indicate that the (−102) orientation is suitable for fast and highly controllable wet-etch patterning. The morphologies of the resulting etched sidewalls on the (−102) surface were dominated by the emergence of flat (001) and (−201) facets. By exploiting the pronounced development of these two facets, we obtained well-defined V-shaped trenches with a crystallographic opening angle of 130.0° when the etching windows were aligned along the [010] direction. In contrast to plasma-based dry etching, where the outcomes often depend heavily on the specific apparatus and processing conditions, we expect this crystallography-driven facet-formation approach to enable the fabrication of highly reproducible V grooves. Given the wide bandgap of β-Ga_2_O_3_, the proposed method is potentially applicable to V-groove-trench metal–oxide–semiconductor field-effect transistors and transmission-type blazed gratings.

## IMPACT STATEMENT

We demonstrate TMAH wet etching on (−102) β-Ga_2_O_3_ with more than tenfold higher rates than standard orientations, enabling robust, crystallographically defined V-grooves for reproducible microfabrication.

## GRAPHICAL ABSTRACT

**Figure:**
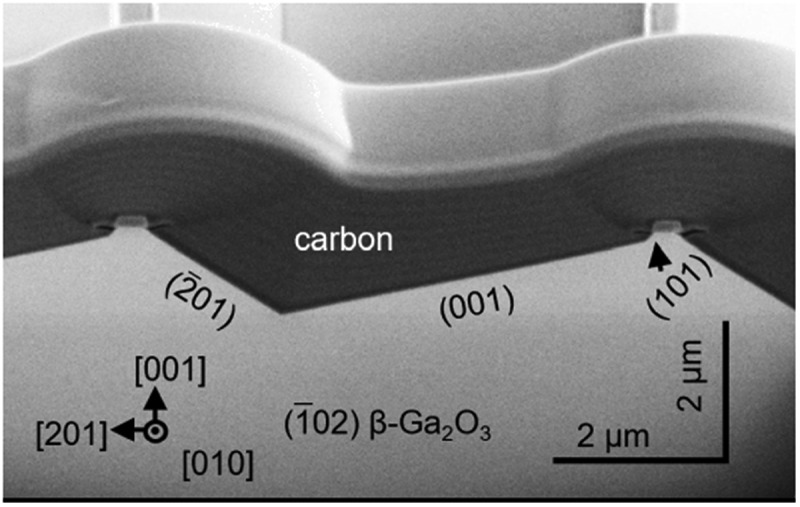

## Introduction

The ultra-wide-bandgap semiconductor, β-Ga_2_O_3_ has attracted significant interest for power-electronic and ultraviolet (UV) optoelectronic applications [1]. Its high critical electric field (approximately 8 MV cm^−1^) [2] enabled by its wide bandgap (≥4.43 eV) [3], results in a Baliga’s figure of merit that is higher than those of conventional power semiconductors such as 4 H-SiC and GaN, indicating that it has potential for applications to next-generation low-loss, high-power electronic devices [2]. In addition, its absorption edge (approximately 280 nm) [3] is close to the solar-blind cutoff, making it suitable for solar-blind deep-UV photodetectors [4]. Moreover, large-area, high-quality wafers can be produced by melt-growth approaches, including edge-defined film-fed growth [5], vertical Bridgman [6], Czochralski [7], floating-zone [8], and oxide-crystal growth using the cold-crucible method [9]. Furthermore, epitaxial layers with high crystalline quality and well-controlled doping can be grown using established techniques, such as molecular-beam epitaxy [10], metal-organic vapor-phase epitaxy [11], and halide vapor-phase epitaxy [12]. Doping can also be implemented using ion implantation, which allows localized donor or deep-acceptor doping [13,14]. The availability of these bulk/epitaxial growth methods, together with appropriate doping processes, provides a practical basis for device fabrication. While the lack of reliable *p*-type doping still constrains device design, a wide range of devices has been reported, including n-type unipolar devices (e.g. Schottky barrier diodes [15–17], trench Schottky barrier diodes [18], metal–semiconductor field-effect transistors [2], metal–oxide–semiconductor field-effect transistors (MOSFETs) [19], and fin field-effect transistors [20]), as well as UV photodetectors (e.g. photoconductors [4], Schottky photodiodes [21], MOS photodiodes [22,23], and avalanche photodiodes [23]). Devices employing alternative p-type oxides such as NiO—for example heterojunction *p*–*n* diodes [24], junction barrier Schottky diodes [25], and super-junction devices [26]—have also been demonstrated, underscoring the material potential of β-Ga_2_O_3_.

Device realization in β-Ga_2_O_3_ depends not only on crystal growth and device design but also on establishing robust fabrication processes. In particular, etching remains a key processing step because the quality of the etched surface and process-induced damage directly affect device performance. Chlorine-based dry etching—specifically, inductively coupled plasma reactive-ion etching using Cl_2_, BCl_3_, and Ar gases [27,28]—is widely used to define device patterns, yet it often causes surface roughening and plasma-induced damage [27–32], making post-dry-etching chemical treatment necessary. As such a post-dry-etching treatment, several comparative studies have suggested that tetramethylammonium hydroxide (TMAH) is a particularly effective wet etchant for surface recovery and damage removal, especially as compared to HF, HCl, H_3_PO_4_, and a sulfuric peroxide mixture [33–36]. In parallel, plasma-free, anisotropic crystallographic-etching approaches based on gas-phase chemistries using a Ga flux (or vapor), triethylgallium, HCl gas, and forming gas [37–44]; wet chemistries using H_3_PO_4_ [45,46]; and photoelectrochemical chemistries using HF/K_2_S_2_O_8_ [47] have been explored as alternatives to conventional plasma-based dry etching.

In this context, we recently demonstrated the efficacy of TMAH-based crystallographic wet etching for fabricating functional structures with precisely defined geometries. Specifically, we achieved atomic-level surface planarization on the (001) plane [48], air-gap formation [49], and improved dry-etched profiles on the (010) plane [50]. During these investigations, we observed that the in-plane side-etching rate on (010) β-Ga_2_O_3_ was significantly enhanced along the [001] direction, which is perpendicular to the ($\bar{\text{1}}$02) plane [49,50]. This finding strongly suggests that the ($\bar{\text{1}}$02) plane exhibits a high wet-etching rate. Although the ($\bar{\text{1}}$02) orientation is not a conventional choice for epitaxial growth or device applications, it corresponds to the {110} planes of the non-primitive face-centered-cubic oxygen sublattice in β-Ga_2_O_3_ and is therefore one of the fundamental planes of the crystal [51]. Furthermore, ($\bar{\text{1}}$02)-oriented substrates are currently emerging as an alternative platform for homoepitaxy [52] and NiO heteroepitaxy [53], selective-area growth, and etching [44,54]. Motivated by these insights and recent developments, in this study we have investigated the crystallographic etching behavior of ($\bar{\text{1}}$02) β-Ga_2_O_3_ using TMAH.

### Experimental methods

We used single-crystal β-Ga_2_O_3_ substrates with various crystallographic orientations—(100), (010), (001), ($\bar{\text{2}}$01), and ($\bar{\text{1}}$02)—for our etching experiments. Except for ($\bar{\text{1}}$02), these orientations are commercially available as standard substrates and are widely used in β-Ga_2_O_3_ studies. The substrate orientation, doping type (Sn-doped or unintentionally Si-doped (UID)), and nominal carrier concentration of these substrates are listed in Table 1.

**Table 1.** Orientation, doping type, and nominal carrier density of the β-Ga_2_O_3_ substrates used in this study.

Orientation	Doping type	Carrier density (cm^−3^)
(100)	Sn-doped	2.2 × 10^18^
(010)	Sn-doped	2.8 × 10^18^
(001)	Sn-doped	6.9 × 10^18^
($\bar{\text{2}}$01)	Sn-doped	2.4 × 10^18^
($\bar{\text{1}}$02)	UID	3.2 × 10^17^
($\bar{\text{1}}$02)	Sn-doped	1.1 × 10^19^

We prepared SiO_2_ etching masks on the β-Ga_2_O_3_ substrates. First, we deposited a SiO_2_ masking layer with a thickness of approximately 0.13 μm using plasma-enhanced chemical-vapor deposition. We then annealed the samples under flowing N_2_ at 800°C for 1 h to densify the SiO_2_ layer and improve its resistance to wet etching [55]. Subsequently, we defined etching windows using laser lithography and opened them using either wet etching with using buffered hydrofluoric (BHF) acid or capacitively coupled plasma reactive-ion etching (CCP-RIE) using CHF_3_ and N_2_ gases. We used BHF acid etching to open square windows (200 μm × 200 μm) for planar etching-rate measurements. We also used CCP-RIE to open circular windows (1.69 μm in diameter), wagon-wheel-patterned windows (1.05 μm in width and 40 μm in length), square windows (200 μm × 200 μm), and striped windows (5.88 μm in window width and 0.32 μm in mask width) to study the TMAH-etched structures.

We then wet-etched the masked β-Ga_2_O_3_ substrates with TMAH using the experimental setup described in our previous paper [50]. We heated a 25 wt% TMAH solution in a polytetrafluoroethylene (PTFE) container with a sealable lid and maintained it at 50°C, 69°C, or 90°C using a hot-plate stirrer. Note that 90°C is the practical upper limit because the boiling point of the solution is 102°C. We monitored the solution temperature using a perfluoroalkoxy alkane (PFA)-coated thermocouple immersed in the solution. After the solution temperature stabilized, we opened the lid, immersed the samples in the solution to initiate wet etching, and closed the lid immediately. After the predetermined etching time (2 h at 50°C, 1.5 h at 69°C, or 1 h at 90°C), we opened the lid and removed the samples from the solution to terminate the etching. We then rinsed the samples with deionized water and dried them with N_2_.

After removing the SiO_2_ mask using BHF acid etching, we measured the resulting TMAH-etched depth using a stylus profilometer. We examined the TMAH-etched structures using scanning electron microscopy (SEM) and atomic force microscopy (AFM) without removing the mask. We also performed cross-sectional SEM observations on cross-sections prepared using focused-ion-beam (FIB) milling, with the top surface protected by a locally deposited carbon layer. The SEM acceleration voltage was set to either 2 or 10 kV. In most cases, we used 2 kV to resolve fine surface features and enhance material contrast. In contrast, we used 10 kV to allow the electron beam to penetrate the SiO_2_ mask and measure the side-etching length, defined as the lateral spacing between the edge of the mask and the etching front in the wagon-wheel-pattern trenches [40].

## Results and discussion

### Orientation dependence of the etching rate

**Figure 1.**
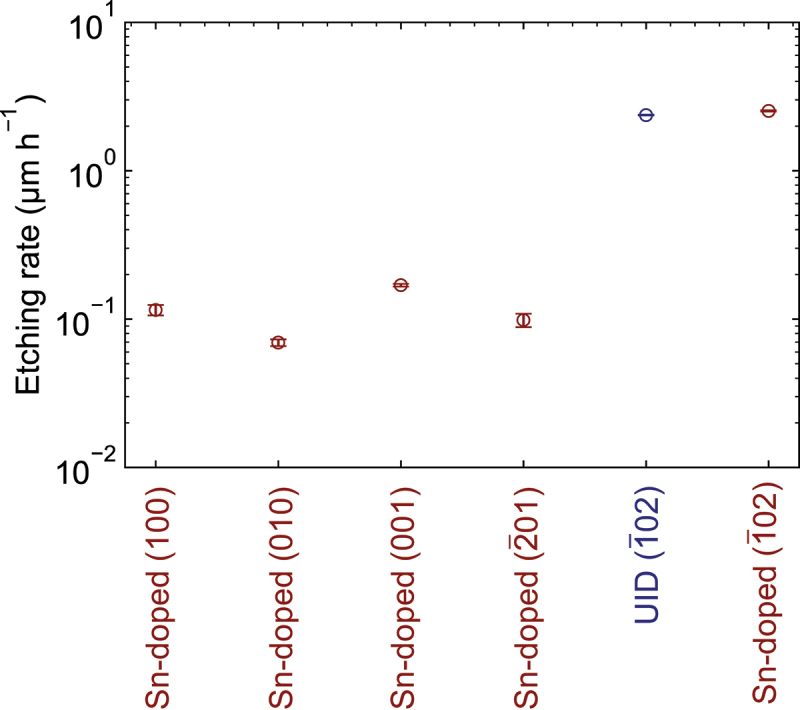
Etching rate of β-Ga_2_O_3_ substrates with various crystal orientations in 25 wt% TMAH at 90°C.

We first compared the planar etching rates of substrates with different orientations after TMAH etching at 90°C for 1 h, as shown in Figure 1. For each orientation, we determined the etching rate by dividing the measured depth by the etching time; we plotted the mean value as a circular symbol, with error bars representing the standard deviation. As expected, we observed a clear difference in the etching rate between the conventionally used (100), (010), (001), and ($\bar{\text{2}}$01) orientations and the ($\bar{\text{1}}$02) orientation. The etching rates of the Sn-doped (100), (010), (001), and ($\bar{\text{2}}$01) substrates were 0.12, 0.07, 0.17, and 0.10 μm h^−1^, respectively, whereas the ($\bar{\text{1}}$02) substrates exhibited the much higher etching rates of 2.37 μm h^−1^ for the UID sample and 2.53 μm h^−1^ for the Sn-doped sample. These results indicate that the etching rate depends strongly on crystal orientation and is only weakly influenced by its carrier concentration.

Possible reasons for the pronounced etching rate of the ($\bar{\text{1}}$02) plane include its high surface-energy density and charge neutrality. The calculated relaxed surface-energy densities are 0.34–0.49 J m^−2^ for (100), 1.67–1.79 J m^−2^ for (010), 1.17 J m^−2^ for (001), 0.96 J m^−2^ for ($\bar{\text{2}}$01), and 1.92 J m^−2^ for ($\bar{\text{1}}$02) [56,57]; the ($\bar{\text{1}}$02) plane thus has the highest surface-energy density among these planes. A higher surface energy generally implies a less-stable surface with a higher density of unsatisfied (dangling) bonds and thus higher chemical reactivity which can facilitate OH^−^-assisted bond breaking in TMAH alkaline solutions [58,59]. Note that this trend is not strictly applicable, especially for the (010) surface, for which the etching rate was lower than those of the (100), (001), and ($\bar{\text{2}}$01) surfaces despite its higher surface-energy density; this is likely because the etched (010) surface is covered with highly etch-resistant facets, as reported in Ref. 49. In addition, the ($\bar{\text{1}}$02) plane forms a charge-neutral layer, in which Ga^3+^ and O^2−^ ions are arranged in a 2:3 ratio with only small vertical displacements (i.e. weak surface rumpling). This charge neutrality and reduced rumpling mitigate the electrostatic repulsion of OH^−^, thereby enhancing adsorption and subsequent dissolution reactions [49,50].

Because its high wet etching rate is suitable for processing, we therefore investigated the etching characteristics of the ($\bar{\text{1}}$02) plane further.

### Temperature dependence of the etching rate

**Figure 2.**
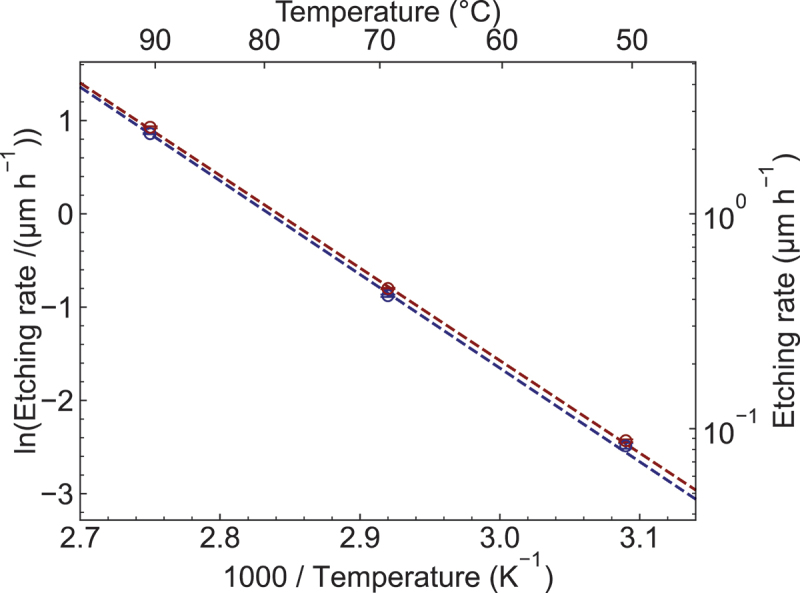
Arrhenius plot of the temperature-dependent etching rate of UID and Sn-doped ($\bar{\text{1}}$02) β-Ga_2_O_3_ substrates in 25 wt% TMAH.

In order to understand the TMAH etching kinetics, we investigated the temperature dependence of the etching rate. Figure 2 is an Arrhenius plot of the TMAH etching rates for the UID and Sn-doped ($\bar{\text{1}}$02) substrates. The linear Arrhenius behavior observed for both samples indicates that, in this temperature range (50°C–90°C), TMAH etching is predominantly surface-reaction-limited rather than mass-transport-limited. Accordingly, the etching rate can be described using the Arrhenius equation:

$$\mathrm{Etching}\;\mathrm{rate}=A\;\exp\left(-\frac{E_{a}}{\mathrm{RT}}\right),$$

where *A* is a pre-exponential factor, *E*_a_ is the activation energy, *R* is the gas constant, and *T* is the absolute temperature. Based on this relationship, *E*_a_ can be determined from a linear fit to the Arrhenius plot. The values we extracted were *E*_a_ = 83.5 kJ mol^−1^ (0.865 eV) for the UID sample and *E*_a_ = 82.5 kJ mol^−1^ (0.855 eV) for the Sn-doped sample. Because these values are nearly identical, *E*_a_ is essentially independent of the carrier concentration. For comparison, the reported values are *E*_a_ = 84.5 and 110 kJ mol^−1^ for H_3_PO_4_ and H_2_SO_4_ etching, respectively, of (100) β-Ga_2_O_3_ [45] and *E*_a_ = 0.49 eV for H_3_PO_4_ etching of (001) β-Ga_2_O_3_ [46].

This clear Arrhenius behavior, with *E*_a_ being almost insensitive to carrier concentration, indicates that the etching rate is predictable and can be readily tuned by adjusting the temperature of the TMAH solution, thereby facilitating process optimization. This controllability, combined with the substantially higher etching rate of the ($\bar{\text{1}}$02) plane compared with other orientations, offers a significant practical advantage for wet-etching processing.

### In-plane anisotropy of the etched structures

Next, we examined the etched structures formed by TMAH etching at 90°C for 1 h to characterize the in-plane etching anisotropy on the ($\bar{\text{1}}$02) plane.

**Figure 3.**
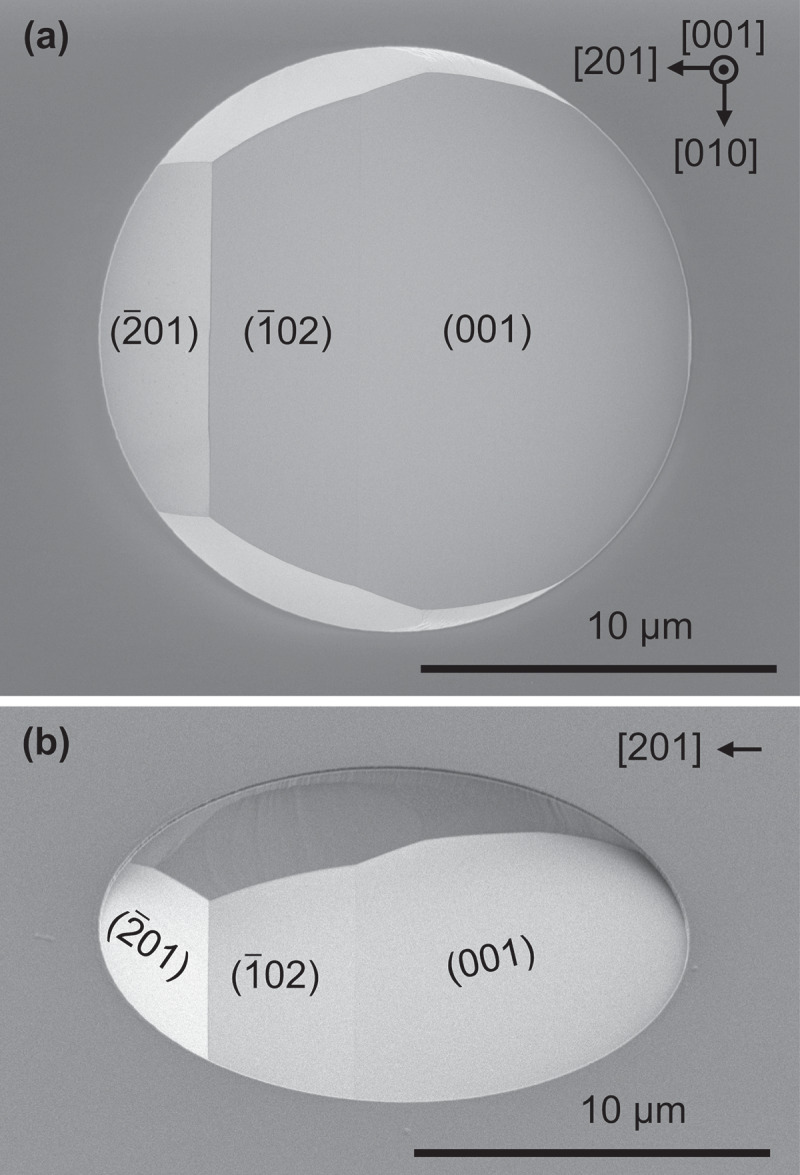
(a) Top-view and (b) 54°-tilted-view SEM images of a TMAH-etched hole formed through a circular window on a ($\bar{\text{1}}$02) β-Ga_2_O_3_ substrate.

First, we used SEM to investigate the etched depression formed through a circular window, as shown in Figure 3. From the top- and oblique-view observations [Figure 3(a,b), respectively], we found that the etched profile was predominantly defined by the emergence of the ($\bar{\text{2}}$01) and (001) sidewall facets that extend continuously from the mask edges on the [201] and [$\bar{\text{2}}$0$\bar{\text{1}}$] sides, respectively. We identified these facets using cross-sectional observations, as described later. The preferential development of the ($\bar{\text{2}}$01) and (001) facets is consistent with their etching resistance being higher than that of the ($\bar{\text{1}}$02) plane [Figure 1]. In contrast, both sidewalls on the [010] and [0$\bar{\text{1}}$0] sides were relatively rough and did not consist of a single facet but exhibited a more complex morphology. Note that we only show the SEM image of the sidewall on the [0$\bar{\text{1}}$0] side [Figure 3(b)] and omit that of the [010] side because the [010] and [0$\bar{\text{1}}$0] directions are crystallographically equivalent.

**Figure 4.**
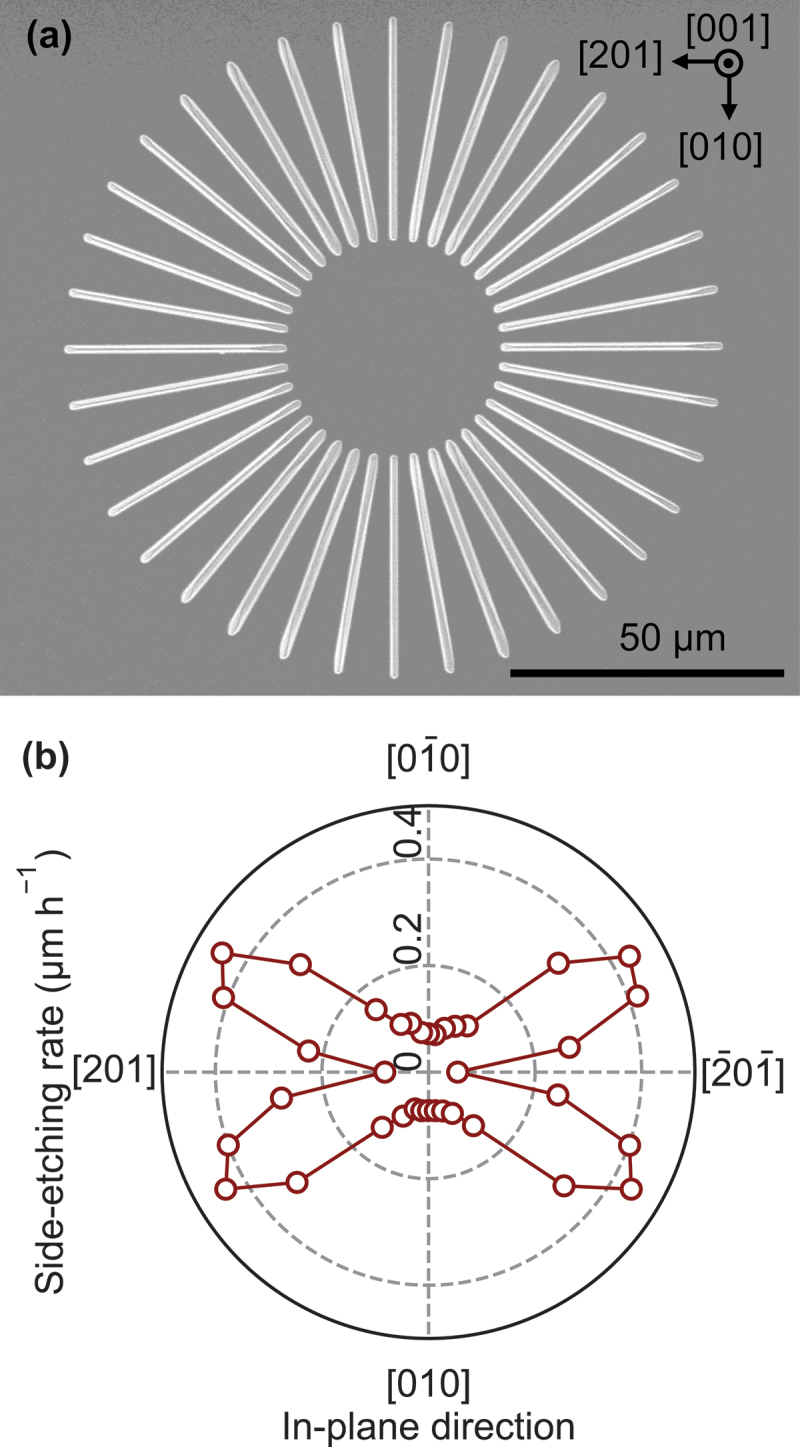
(a) Top-view SEM image of TMAH-etched trenches formed through wagon-wheel patterned windows on the ($\bar{\text{1}}$02) β-Ga_2_O_3_ substrate. (b) Polar plot of the side-etching rate extracted from (a).

Next, we evaluated the in-plane dependence of the side-etching rate using wagon-wheel-patterned trenches, as shown in Figure 4. Here, the wagon-wheel pattern included trenches aligned along the [201] and [010] crystallographic directions. We measured the side-etching lengths shown in Figure 4(a) using SEM observations at the high accelerating voltage of 10 kV; the corresponding side-etching rates are summarized in the polar plot in Figure 4(b). The pattern of the plot reflects the crystallographic symmetry of β-Ga_2_O_3_; the upper-half pattern on the [0$\bar{\text{1}}$0] side was nearly identical to the lower-half pattern on the [010] side, which is consistent with the mirror symmetry across the (010) plane. Also, the left-half pattern on the [201] side was similar to the right-half pattern on the [$\bar{\text{2}}$0$\bar{\text{1}}$] side, which is compatible with the twofold rotational symmetry about the [010] axis. In addition, the polar plot exhibits four local minima – in the [010], [0$\bar{\text{1}}$0], [201], and [$\bar{\text{2}}$0$\bar{\text{1}}$] directions – which suggests the development of low-etching-rate sidewall facets, including the (010), ($\bar{\text{2}}$01), and (001) facets. These in-plane directions are particularly interesting because they suppress undercut etching and improve mask-defined pattern fidelity.

**Figure 5.**
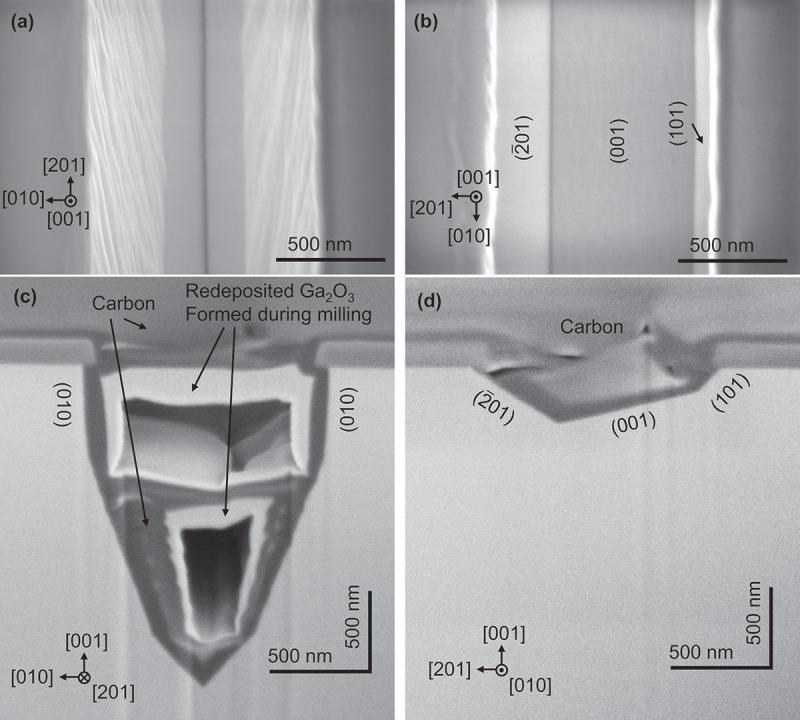
**(*Two columns*)** (a) and (b) Top-view SEM images of TMAH-etched trenches aligned along the [201] and [010] directions, respectively, in the wagon-wheel pattern shown in Figure 4(a). Panels (c) and (d) show 54°-tilted-view cross-sectional SEM images of the corresponding trenches in (a) and (b), respectively.

We, therefore, examined the TMAH-etched trenches oriented along the [201] and [010] directions in the wagon-wheel pattern further, as shown in Figure 5. Figure 5(a,c), respectively, show top-view and oblique-view cross-sectional SEM images of the [201]-oriented trench, whereas Figure 5(b,d), respectively, show the corresponding images for the [010]-oriented trench.

The [201]-oriented trench exhibited a bilaterally symmetric but relatively complex sidewall profile, consisting of a vertical (010) plane followed by tilted rough and smooth planes from top to bottom [Figure 5(a,c)]. Because of its vertical orientation, we were not able to evaluate the roughness of the (010) plane solely from these images. We also were not able to evaluate the tilted rough planes at the middle section uniquely, as the measured angle of 33.6° between the planes fell between the 29.4° angle that corresponds to the (1$\bar{\text{4}}\;\bar{\text{2}}$)/(14$\bar{\text{2}}$) pair and the 38.5° angle of the (1$\bar{\text{3}}\;\bar{\text{2}}$)/(13$\bar{\text{2}}$) pair. Similarly, we were unable to identify the tilted smooth planes at the bottom uniquely, as the measured opening angle of 77.0° fell between the 55.3° angle that corresponds to the (1$\bar{\text{2}}\;\bar{\text{2}}$)/(12$\bar{\text{2}}$) pair and the 92.7° of the (1$\bar{\text{1}}\;\bar{\text{2}}$)/(11$\bar{\text{2}}$) pair. These discordances suggest a combination of multiple stable planes or transitional facet formation during the etching process.

On the other hand, the [010]-oriented trench exhibited an asymmetric but simpler profile that consists of smooth ($\bar{\text{2}}$01), (001), and (101) facets [Figure 5(b,d)]. Although the etching rate of the (101) plane was not directly quantified because this orientation is not included in the standard set of substrate orientations, its prominent appearance suggests a low etching rate. This is compatible with its nature as an oxygen-close-packed plane, sharing similar structural characteristics with the ($\bar{\text{2}}$01) plane [51]. Note that the trench profile was completely bounded by slow-etching ($\bar{\text{2}}$01), (001), and (101) facets—particularly the ($\bar{\text{2}}$01) and (001) facets—consistent with the results from the observation of the etched depression beneath the circular window (Figure 3).

**Figure 6.**
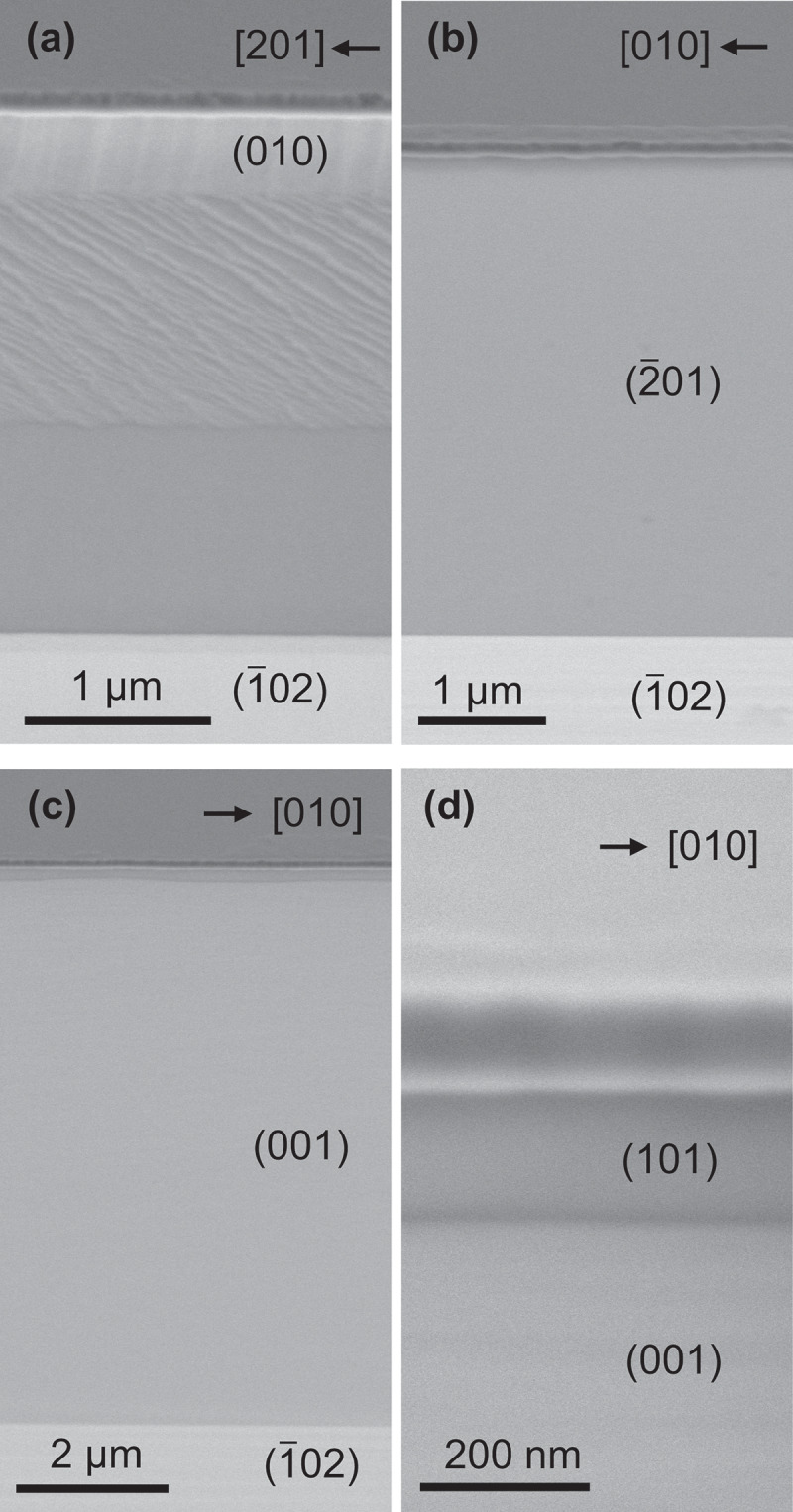
SEM images at a 54°-tilted-view of the sidewalls of the TMAH-etched depression formed through a 200 × 200 μm square window, the sides of which are parallel to the [201] and [010] directions. The sidewalls corresponding to the [201] side are shown in (a), whereas those corresponding to the [010] side are shown in (b)–(d). Panels (c) and (d) are the opposite sides of (b). Panel (d) is a higher magnification image of (c).

We also observed the sidewall morphologies of the corresponding TMAH-etched structures formed through a larger square window, for which the mask edges were aligned along the [201] and [010] directions, as shown in Figure 6. These tilted-view SEM images reveal the morphologies of the (010) and (101) sidewalls, which are completely and partially obscured in Figure 5(a,b), respectively. The (010) surface was relatively rough [Figure 6(a)], in agreement with our previous results for TMAH etching on the (010)-oriented substrates [49,50]. We confirmed that the (101) facet is smooth [Figure 6(d)], and we reconfirmed that the ($\bar{\text{2}}$01) and (001) facets are smooth [Figures 6(b,c)].

**Figure 7.**
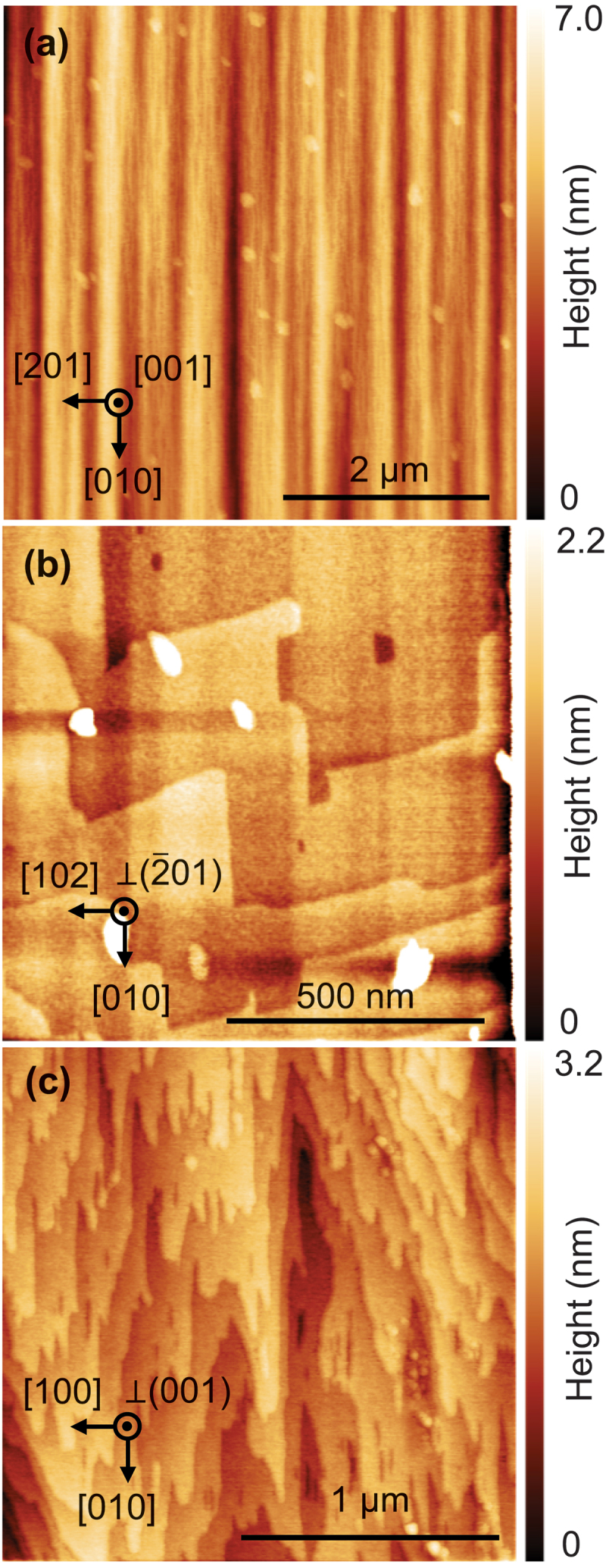
AFM images of (a) the planar ($\bar{\text{1}}$02) surface, (b) the inclined ($\bar{\text{2}}$01) facet, and (c) the inclined (001) facet of the TMAH-etched depression.

We further performed AFM on the ($\bar{\text{1}}$02), ($\bar{\text{2}}$01) and (001) surfaces of the depression shown in Figure 6. Figure 7(a,c) represent the surface morphologies of the planar ($\bar{\text{1}}$02) surface and the inclined ($\bar{\text{2}}$01) and (001) facets, respectively. We were not able to evaluate the inclined (101) facet using AFM because its exposed area was too small for measurement. The other inclined ($\bar{\text{2}}$01) and (001) facets—particularly the ($\bar{\text{2}}$01) facet—provide only limited areas for AFM observations; therefore, the scan areas for the ($\bar{\text{2}}$01) and (001) facets were smaller than that for the ($\bar{\text{1}}$02) plane. Step-and-terrace structures are clearly observed on these facets, which is consistent with their mode of development [Figure 7(b,c), respectively]. In contrast, the surface morphology of the ($\bar{\text{1}}$02) plane was relatively rough and consisted of [010]-elongated structures [Figure 7(a)]. Although the origin of the several-nanometer-high particles observed on the ($\bar{\text{2}}$01) facet is unclear at the moment, they may be related to the relatively low etching rate of this facet. Further investigation is required to clarify the origin of these particles and to identify methods for preventing their formation. The root-mean-square roughness values of the ($\bar{\text{1}}$02), ($\bar{\text{2}}$01), and (001) surfaces were 0.89 nm, 0.61 nm with particles (0.22 nm without particles), and 0.39 nm, respectively.

These findings indicate that trenches oriented along the [010] direction are particularly advantageous for TMAH etching on ($\bar{\text{1}}$02) β-Ga_2_O_3_. In this orientation, side etching is strongly suppressed because the trench is bounded by well-developed ($\bar{\text{2}}$01), (001), and (101) facets, yielding a profile consistently defined by crystallographic faceting. The resulting geometry—an asymmetric V groove—should therefore be highly reproducible and only weakly sensitive to minor fluctuations in the TMAH etching conditions.

### Formation of asymmetric V-shaped grooves

**Figure 8.**
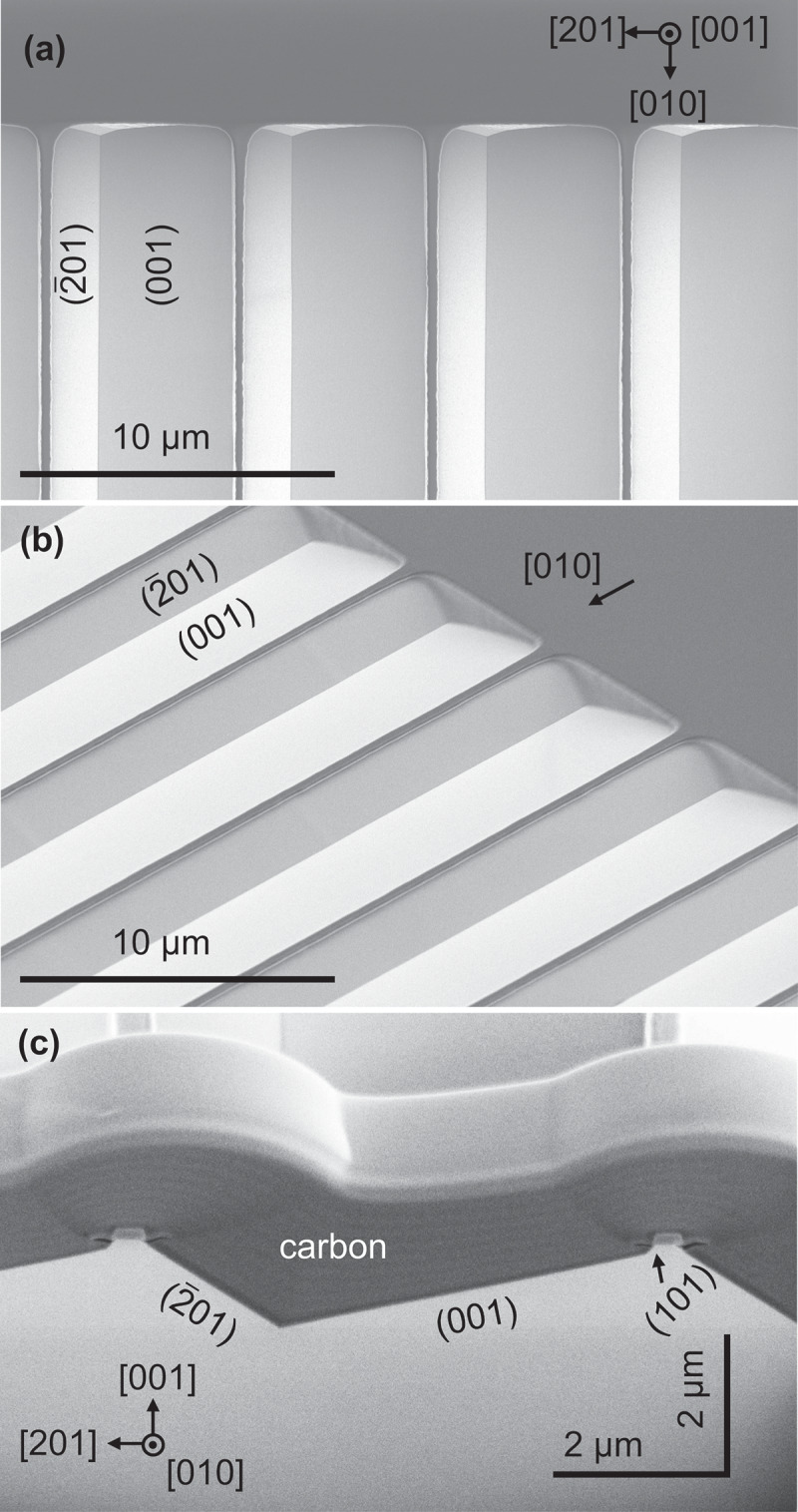
(a) Top-view, (b) 54°-tilted-view, and (c) 54°-tilted-view cross-sectional SEM images of TMAH-etched V grooves formed through etching windows aligned along the [010] direction on the ($\bar{\text{1}}$02) β-Ga_2_O_3_ substrate.

Finally, we used TMAH etching at 90°C for 1 h to demonstrate the formation of a V-groove array through striped windows oriented along the [010] direction, as shown in Figure 8. To maximize the V-groove area with a view toward blazed-grating applications, we reduced the striped mask to the minimum achievable with optical lithography [Figure 8(a)], and we set the width of the etching window to be larger than that used in forming the wagon-wheel pattern. The resulting V grooves were primarily defined by flat ($\bar{\text{2}}$01) and (001) facets, with only a negligible contribution from the (101) facet [Figures 8(a–c)]. This contrasts with the result for the narrower etching window in the wagon-wheel pattern [Figure 6(d)], which suggests that the formation of the (101) facet is suppressed near the mask edge on the [$\bar{\text{2}}$0$\bar{\text{1}}$] side. We measured the opening angle between the ($\bar{\text{2}}$01) and (001) facets to be 128.5°, which is in good agreement with the corresponding crystallographic angle (130.0°).

Given the high UV optical transparency and excellent power-device figure of merit enabled by the wide bandgap of β-Ga_2_O_3_, these V grooves on ($\bar{\text{1}}$02) β-Ga_2_O_3_ are particularly attractive for UV-transparent molds for the UV-nanoimprint-lithography fabrication of blazed gratings, transmission-type blazed gratings [60] capable of operating down to the UV-B range, and V-groove trench MOSFETs [61,62], although this study does not specifically aim at these applications.

## Summary

We systematically investigated the wet etching of ($\bar{\text{1}}$02) β-Ga_2_O_3_ in heated 25 wt% TMAH. The planar etching rate was an order of magnitude higher than those of the widely used (100), (010), (001), ($\bar{\text{2}}$01) orientations, possibly due to the high surface energy and charge neutrality of the ($\bar{\text{1}}$02) plane. The etching rates measured at 50°C–90°C followed the Arrhenius relation, indicating surface-reaction-limited kinetics in this range. These results demonstrate the suitability of the ($\bar{\text{1}}$02) orientation for wet-etch patterning. Systematic evaluations using various mask geometries further showed that [010]-oriented windows enable selective-area etching with negligible side etching and produce facet-defined asymmetric V-grooves bounded mainly by flat ($\bar{\text{2}}$01) and (001) planes. Such damage-free, highly reproducible facet surfaces are difficult to achieve by dry etching.
